# A limited role for p53 in modulating the immediate phenotype of Apc loss in the intestine

**DOI:** 10.1186/1471-2407-8-162

**Published:** 2008-06-05

**Authors:** Karen R Reed, Valerie S Meniel, Victoria Marsh, Alicia Cole, Owen J Sansom, Alan R Clarke

**Affiliations:** 1 Cardiff School of Biosciences, Cardiff University, Cardiff, CF10 3US, UK; 2 The Beatson Institute, Garscube Estate, Glasgow, G61 1BD, UK

## Abstract

**Background:**

p53 is an important tumour suppressor with a known role in the later stages of colorectal cancer, but its relevance to the early stages of neoplastic initiation remains somewhat unclear. Although p53-dependent regulation of Wnt signalling activity is known to occur, the importance of these regulatory mechanisms during the early stages of intestinal neoplasia has not been demonstrated.

**Methods:**

We have conditionally deleted the Adenomatous Polyposis coli gene (*Apc*) from the adult murine intestine in wild type and p53 deficient environments and subsequently compared the phenotype and transcriptome profiles in both genotypes.

**Results:**

Expression of p53 was shown to be elevated following the conditional deletion of Apc in the adult small intestine. Furthermore, p53 status was shown to impact on the transcription profile observed following Apc loss. A number of key Wnt pathway components and targets were altered in the p53 deficient environment. However, the aberrant phenotype observed following loss of *Apc *(rapid nuclear localisation of β-catenin, increased levels of DNA damage, nuclear atypia, perturbed cell death, proliferation, differentiation and migration) was not significantly altered by the absence of p53.

**Conclusion:**

p53 related feedback mechanisms regulating Wnt signalling activity are present in the intestine, and become activated following loss of Apc. However, the physiological Wnt pathway regulation by p53 appears to be overwhelmed by Apc loss and consequently the activity of these regulatory mechanisms is not sufficient to modulate the immediate phenotypes seen following Apc loss. Thus we are able to provide an explanation to the apparent contradiction that, despite having a Wnt regulatory capacity, p53 loss is not associated with early lesion development.

## Background

p53 is implicated in colorectal cancer as the final step of multi-step carcinogenesis leading to carcinoma formation [1]. However, p53 is also postulated to be involved as a critical negative regulator of the Wnt signalling pathway, arguing that it should also have a role in the tumour initiation process. To date, crossing p53 deficient mice to the *Apc*^*min *^mouse has variably been reported to have no effect or to modify adenoma formation [2-4]. Consequently there is a need to further clarify the role of p53 function in intestinal neoplasia.

Increased levels of β-catenin lead to increased transcription of *p14ARF *(in human cells, *p19ARF *in murine cells) which in turn inactivates Mdm2, allowing the activation of p53 [5]. Active p53 is then able to down-regulate *β-catenin *and a number of mechanisms for this feedback have been proposed. These include a GSK3β independent mechanism where the p53 target gene *Siah1 *interacts with APC to promote the degradation of β-catenin [6]. A GSK3β, CK1 dependent mechanism has also been proposed, wherein, p53 activity mediates a faster mobilization of Axin into the degradation complex. This results in enhanced phosphorylation of β-catenin on key NH2-terminal serines and consequently a heightened β-catenin turnover [7]. In both of these scenarios there is, presumably, a requirement for active Apc in order to obtain inhibition of Wnt signalling. However, p53 activity also affects the expression of genes that are known regulators of Wnt signalling e.g. p53 induces *Dkk1 *expression which is an inhibitor of the Wnt signalling pathway acting upstream of β-catenin [8], while *Tcf-4 *mRNA is down-regulated after increased p53 expression [9]. Thus, in these scenarios Wnt signalling could be inhibited independently to loss of *Apc*.

Here, we report the consequence of p53 deficiency upon the phenotypes observed immediately following *Apc *loss. These data clarify the extent to which p53-dependent inhibition of Wnt signalling can occur in this setting, and also indicate whether p53 status is relevant to the tumour initiation process. To achieve this, we utilised mice bearing a conditional Apc allele on either a wild type or p53 deficient background [10,11] and determined if any p53 dependent feedback loops have a functional consequence immediately following Apc loss. Thus we are able to address whether p53 contributes to the early stages of intestinal neoplasia or whether its role is restricted to the latter stages of carcinogenesis as previously proposed [1].

## Methods

All experiments were performed according to UK Home Office regulations. Induction of Apc loss was initiated by giving daily injections (i.p.) of β-napthoflavone (80 mg/kg). Tissues were harvested at the appropriate time point and processed as previously described [11]. Immunohistochemistry (IHC) was performed on paraffin embedded tissues fixed in 4% formaldehyde at 4°C for no more than 24 hours prior to processing. The following antibodies were used for IHC; p53 1:50, MS104 Labvision, β-catenin 1:50, C19220, Transduction Laboratories, cMyc 1:50 sc-764, Santa Cruz, p21CIP1/WAF1 1:500, Santa Cruz Technologies, γ H2AX 1:300, JBW 301, Upstate, lysozyme 1:200, A0099 Dako. Grimelius staining was performed as previously described by Sansom *et al*. 2004. Apoptosis and mitotic index were scored from H&E stained sections as previously described [12] and apoptosis was independently confirmed by IHC staining with an antibody against active caspase 3 (1:750; R&D systems data not shown). Nuclear area was characterised from H&E stained sections using the AnalySIS (Soft Imaging Systems) software. Primers for quantitative real-time RT-PCR (QRT-PCR) (listed in additional file 1) were designed using primer3 to span intron-exon boundaries wherever possible. QRT-PCR reactions were performed using a chromo4 real-time detector (MJ Research) and standard protocols of 60°C annealing temperature. Triplicate samples from each genotype were amplified in duplicate and the expression levels of the genes of interest were normalised to the housekeeping control gene *β-actin *and analysed as previously described [13]. Affymetrix Gene Arrays were run at the Cancer Research UK facility at the Paterson Institute for Cancer Research, UK using MOE430_2 chips and triplicate intestinal samples for each genotype 5 days post induction using Cre recombinase; *Cre+Apc*^*fl*/*fl*^*p53*^+/+^,*Cre+Apc*^*fl*/*fl*^*p53*^-/-^, *Cre+Apc*^+/+^*p53*^+/+ ^and *Cre+Apc*^+/+^*p53*^-/-^. The array data generated is available from the Affymetrix MIAMEVICE web page [14].

## Results and discussion

### P53 expression and regulation following the loss of Apc

**Figure 1:**
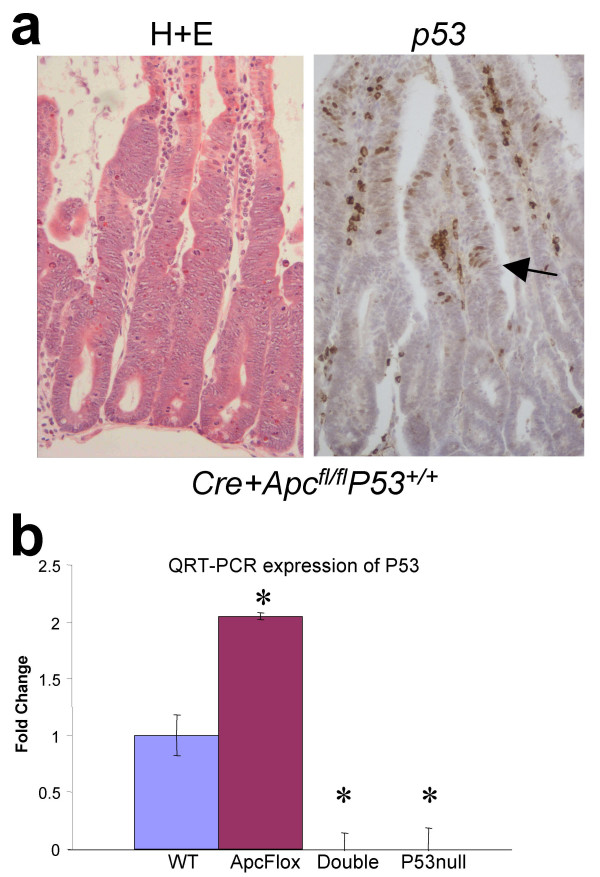
**Characterisation of p53 expression levels in induced *Cre+Apc*^*fl*/*fl*^*p53*^+/+ ^intestinal samples**. a) p53 IHC was performed 4 days post Apc loss and shows that p53 is only up-regulated in a subset of cells, towards the leading edge but within the aberrant structure seen following Apc loss. b) QRT-PCR expression analysis of intestinal samples 4 days post Apc loss (ApcFlox) confirms that *p53 *transcript is up-regulated approximately 2 fold.

**Figure 2:**
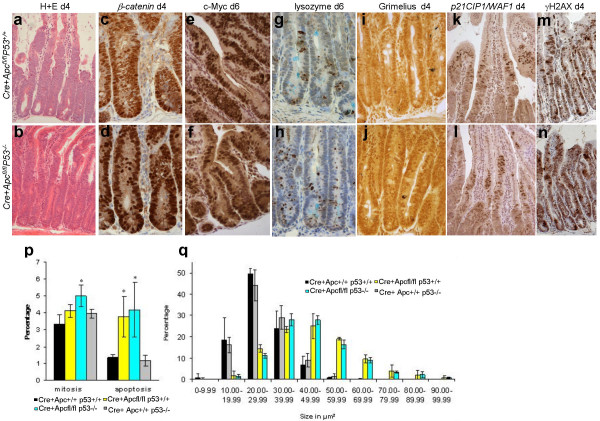
**Phenotypic characterisation of induced *Cre+Apc*^*fl*/*fl*^*p53*^-/- ^and *Cre+Apc*^*fl*/*fl*^*p53*^+/+ ^intestinal samples**. The top panel represents induced *Cre+Apc*^*fl*/*fl*^*p53*^+/+ ^intestinal samples, while the bottom panel represents induced *Cre+Apc*^*fl*/*fl*^*p53*^-/- ^intestinal samples. a+b) H&E staining displays no ascertainable difference between (a) the induced *Cre+Apc*^*fl*/*fl*^*p53*^+/+ ^and (b) the induced *Cre+Apc*^*fl*/*fl*^*p53*^-/-^. c+d) β-catenin IHC and e+f) cMyc IHC display nuclear staining throughout the aberrant structure in both sample types. g+h) IHC detecting lysozyme positivity shows that paneth cell mis-localisation occurs in an identical manner in both genotypes with complete mis-localisation occurring 6 days after induction. i+j) Grimelius staining shows no ascertainable difference between the two induced genotypes. k+l) p21CIP1/WAF1 IHC displays a band of positively stained cells displaying an enlarged nuclear area and the leading edge of aberrancy adjacent to phenotypically normal cells in both induced genotypes. m+n) γ H2AX IHC shows no difference between the 2 genotypes. p) Apoptotic and mitotic crypt indices show no differences between the induced *Cre+Apc*^*fl*/*fl*^*p53*^-/- ^and *Cre+Apc*^*fl*/*fl*^*p53*^+/+ ^intestinal samples (assessed using t-test), asterisks signify a significant difference (p < 0.05) compared to the control levels. q) Characterisation of the nuclear area at the crypt-villus (or aberrant-normal) junction show both the induced *Cre+Apc*^*fl*/*fl*^*p53*^-/- ^and *Cre+Apc*^*fl*/*fl*^*p53*^+/+ ^samples display an increase in the number of larger nuclei compared to control *Cre+Apc*^+/+^*p53*^-/- ^and *Cre+Apc*^+/+^*p53*^+/+ ^samples. Yet again no differences between the induced *Cre+Apc*^*fl*/*fl*^*p53*^-/- ^and *Cre+Apc*^*fl*/*fl*^*p53*^+/+ ^samples could be identified.

Given that we expect to see p53 expression up-regulated following activation of Wnt signalling, we initially characterised the p53 expression profile in intestines following the conditional deletion of Apc. We have developed a robust protocol which utilises the Ah-cre transgene (possessing an inducible Cyp1a-driven Cre recombinase [15]) in combination with loxP flanked Apc alleles [16] allowing us to conditionally delete Apc in the adult small intestine [11]. We have previously demonstrated that such conditional deletion of Apc results in rapid nuclear localisation of β-catenin, acute activation of Wnt signalling and perturbed cell death, proliferation, differentiation and migration [11]. We have also previously performed affymetrix microarray expression analysis comparing *Cre+Apc*^*fl*/*fl*^*p53*^+/+ ^and *Cre+Apc*^+/+^*p53*^+/+ ^intestines [11], and this data suggested an approximately 1.7 fold up-regulation of *p53 *expression following the loss of Apc. We have now verified this finding by both quantitative real-time RT-PCR (QRT-PCR) (Figure 1) and immunohistochemistry (IHC) (Figure 1). IHC analysis demonstrated that elevated p53 protein occurred only in a discrete set of cells towards the leading edge, but still within, the aberrant structure seen following the loss of Apc (Figure 1) and was not global as was initially expected. Nuclear p53 protein is not seen within the intestinal epithelium of induced control samples (*Cre+LacZ+Apc*^+/+^*p53*^+/+^) (additional file 2). Thus we are able to exclude the possibility that p53 activation is occurring as a consequence of DNA breaks transiently generated during Cre mediated recombination (recombination occurs around LoxP flanked stop cassette upstream of the LacZ allele). It is pertinent to note that widespread nuclear β-catenin and c-Myc occurs following Apc loss (Figure 2c+e) resulting in activation of Wnt target genes throughout the aberrant structure [11]. The restricted pattern p53 expression suggests that p53 is not a global Wnt target gene, or indeed a global c-Myc target gene, but is deregulated as a consequence of Apc loss in conjunction with other factors.

### Characterisation of p53 deficient Apc deficient intestines

In order to assess the importance of p53 status upon the early consequences of Apc loss we generated p53 null mice possessing the Ah-cre transgene and loxP flanked Apc alleles (designated *cre+Apc*^*fl*/*fl*^*p53*^-/- ^mice) and conditionally deleted Apc in a p53 deficient environment. Characterisation of induced *cre+Apc*^*fl*/*fl*^*p53*^-/- ^mice demonstrated that the additional loss of p53 in the context of Apc deficiency did not alter the gross phenotype observed following the loss of Apc (Figure 2).

Induced *cre+Apc*^*fl*/*fl*^*p53*^-/- ^mice displayed an aberrant crypt-villus architecture possessing an expanded area of morphologically atypical "crypt-like" cells evident on hematoxylin-and-eosin-stained material (Figure 2a+b), in a manner indistinguishable from *Apc *loss alone. Rapid nuclear localisation of β-catenin also occurred in the aberrant areas in both genotypes (Figure 2c+d) and the time scale for loss of differentiation status of enteroendocrine cells (assessed by Grimelius staining) and Paneth and goblet cell mislocalisation (assessed using anti-Lysozyme IHC and alcian blue staining respectively) was identical in both genotypes (Figure 2g–j).

Apc loss (in induced *cre+Apc*^*fl*/*fl*^*p53*^+/+ ^mice) is known to result in an increase in nuclear area primarily in the cells towards the leading edge of the aberrant structure [17]. The precise mechanisms and relevance of this finding is currently not known, but given that up-regulation of p53 occurs within this area, we quantified nuclear area in *cre+Apc*^*fl*/*fl*^*p53*^-/- ^samples to determine if p53 played any role in this phenotype. Several lines of evidence suggest such a role for p53. For example, p53-dependent G1/S arrest or G2/M arrest are mechanisms employed to prevent genomically unstable cells progressing [18-21]. Furthermore, p53 is up-regulated in response to DNA damage and other stimuli that can result in aneuploidy, with p53-deficient cells failing to arrest and developing centrosome and spindle dysfunction and ultimately aneuploidy [22,23]. Thus it might be expected that p53 deficiency within our system might result in elevated levels of nuclear atypia. However, no difference was observed in nuclear area within the leading edge of aberrant morphology between induced *cre+Apc*^*fl*/*fl*^*p53*^+/+ ^and *cre+Apc*^*fl*/*fl*^*p53*^-/- ^intestines (Figure 2q), and this phenomenon is therefore independent of p53 status. Furthermore, IHC analysis demonstrated continued up-regulation of the p53-dependent target gene p21CIP1/WAF1 in the absence of p53 (Figure 2k+l), which is considered to act as a marker of polyploidy [24].

Given that p53 is a key gene in signalling DNA damage induced apoptosis and cell cycle arrest in the murine small intestine, we also assessed whether the apoptosis that is induced following Apc loss was dependent on p53. This is particularly relevant given our recent data showing that apoptosis induced by Apc loss is dependent on the proto-oncogene c-Myc [25] and recent publications highlighting the importance of oncogenic activation of DNA checkpoints at the earliest stages of tumourigenesis [26,27]. Importantly the levels of apoptosis were not affected by p53 deficiency (Figure 2p), proving that p53 is not required for the apoptotic response seen in this setting. Furthermore, there was no significant difference in the mitotic index between induced *cre+Apc*^*fl*/*fl*^*p53*^+/+ ^and *cre+Apc*^*fl*/*fl*^*p53*^-/- ^intestines (Figure 2p) and we believe that p53 is not required for the increase in mitotic index seen following Apc loss. That said induced *cre+Apc*^*fl*/*fl*^*p53*^-/- ^intestines were significantly elevated compared to wild type while induced *cre+Apc*^*fl*/*fl*^*p53*^+/+ ^intestines were not (Figure 2p). Therefore, there is a trend based solely on the mean levels of mitosis following induction, which suggests p53 may act to repress mitosis following Apc loss. However, we consider the evidence to support such a p53 dependent role to be rather weak.

**Figure 3:**
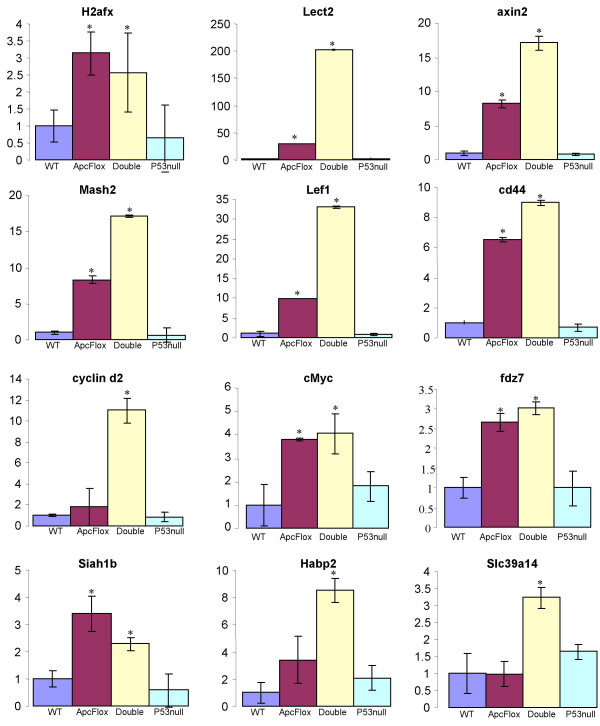
**QRT-PCR expression analysis of candidate genes**. QRT-PCR expression analysis was on intestinal samples from *Cre+Apc*^*fl*/*fl*^*p53*^+/+ ^(ApcFlox),*Cre+Apc*^*fl*/*fl*^*p53*^-/- ^(Double), *Cre+Apc*^+/+^*p53*^+/+ ^(WT) and *Cre+Apc*^+/+^*p53*^-/- ^(P53null) intestinal samples, 4 days post induction. The histogram shows the fold change of expression levels in experimental samples relative to control samples, while error bars show the standard deviation of ΔC_T _values obtained for the replicates of each sample type. Asterisks denote a significant difference between the experimental and control ΔC_T _values, as assessed using the T-test p < 0.05.

We also assessed *γ*-H2AX levels to investigate the levels of DNA damage occurring in both genotypes. *γ*-H2AX foci have been shown to localize at double-strand DNA breaks [28] and we show here that expression levels of *γ-H2AX *are up-regulated (approximately 3 fold) following Apc loss (Figure 3) and widespread γ-H2AX staining occurs throughout the aberrant structure (Figure 2m) which is not seen in induced *cre+Apc*^+/+^*P53*^+/+ ^control samples (data not shown). This widespread γ-H2AX staining is seen at a similar level in the absence of p53 (Figure 2n and QRT-PCR analysis confirms that expression levels are not altered in the absence of p53 (Figure 3). Thus, loss of Apc results in the rapid elevation of γ-H2AX levels, consistent with its role as a marker of increased DNA damage, another hallmark of neoplasia which we observe to be independent of p53 status.

### Characterisation of Wnt signalling in a p53 deficient Apc deficient intestine

We next wished to closely address whether p53 loss was subtly affecting Wnt signalling and therefore employed affymetrix micro-array analysis to compare the transcriptome profiles of induced *Cre+Apc*^*fl*/*fl*^*p53*^-/- ^and induced *Cre+Apc*^*fl*/*fl*^*p53*^+/+ ^intestines compared to control *Cre+Apc*^+/+^*p53*^+/+^.

Array date was screened (using a cut-off of at least one p value for each probe ID <0.2), normalised (global geometric mean) using the MaxD/View-Program and analysed using the SAM-Program [29,30]. Pair-wise comparison between induced *Cre+Apc*^*fl*/*fl*^*p53*^-/- ^and control induced *Cre+Apc*^+/+^*p53*^+/+ ^samples identified a total of 78 significantly altered probe ID's using a stringent 2% false discovery rate (FDR) (additional file 3). Of these, 53 (Table 1) were not identified in SAM outputs comparing induced *Cre+Apc*^*fl*/*fl*^*p53*^+/+ ^to induced control *Cre+Apc*^+/+^*p53*^+/+ ^samples or induced *Cre+Apc*^+/+^*p53*^-/- ^to induced control *Cre+Apc*^+/+^*p53*^+/+ ^samples. These gene changes are therefore classified as statistically altered (using a 2% FDR cut-off) specifically following the combined loss of both p53 and Apc. It should be noted that a proportion of these genes may display an altered expression profile in either induced *Cre+Apc*^*fl*/*fl*^*p53*^+/+ ^or induced *Cre+Apc*^+/+^*p53*^-/-^, but they are not identified as statistically altered by SAM analysis using the stringent cut-off level of 2%FRD, but are only identified using less stringent cut-off levels. Two of these genes, Lect2 and Axin2, are known Wnt target genes, the expression profiles of which were confirmed across the genotypes using Q-RTPCR (Figure 3). Indeed for both genes, although they are deregulated following *Apc *loss, we observed heightened induction in the combined absence of Apc and p53. This is consistent with loss of a feedback mechanism in the absence of p53, or indeed the induction of an alternate feedback mechanism, as both Axin2 and Lect2 are known Wnt repressors as well as Wnt targets [31,32].

**Table 1:** Changes unique tox double *Cre+Apc*^*fl*/*fl*^*p53*^-/- ^samples.

**Probe set ID**	**Gene Symbol**	**Gene Title**	**Fold Change in Apc^*fl*/*fl *^p53^-/- ^v WT**
1418368_at	Retnlb	resistin like beta	0.002
1422422_at	Defcr4	defensin related cryptdin 4	27.73
1425221_at	Agr3	anterior gradient homolog 3 (Xenopus laevis)	27.20
1460582_x_at	a	Nonagouti	17.82
1421837_at	Rps18	ribosomal protein S18	8.12
1422934_x_at	Defcr-rs7	defensin related cryptdin, related sequence 7	30.91
452183_a_at	Gtl2	GTL2, imprinted maternally expressed untranslated mRNA	25.82
1421838_at	Rps18	ribosomal protein S18	8.60
1459443_at	Kcnq1	Potassium voltage-gated channel, subfamily Q, member 1	9.67
1447260_at	ENSMUSG000000734	ENSMUSG00000073403	12.11
1426340_at	Slc1a3	solute carrier family 1 (glial high affinity glutamate transporter), member 3	13.83
1455274_at	a	Nonagouti	19.31
1422071_at	Lgals6	lectin, galactose binding, soluble 6	11.52
1420748_a_at	Adat1	adenosine deaminase, tRNA-specific 1	4.33
1423017_a_at	Il1rn	interleukin 1 receptor antagonist	12.54
1449492_a_at	Lect2	leukocyte cell-derived chemotaxin 2	43.73
1443536_at	Slc7a11	solute carrier family 7 (cationic amino acid transporter, y+ system), member 11	13.16
1446728_at	D3Ertd797e	DNA segment, Chr 3, ERATO Doi 797, expressed	2.39
1459779_s_at	Setd8	SET domain containing (lysine methyltransferase) 8	6.92
1421257_at	Pigb	phosphatidylinositol glycan anchor biosynthesis, class B	11.65
1446730_at	C79267	Expressed sequence C79267	10.65
1459834_x_at	---	---	4.64
1460604_at	Cybrd1	cytochrome b reductase 1	22.55
1427580_a_at	Rian	RNA imprinted and accumulated in nucleus	9.54
1418708_at	Apoc4	apolipoprotein C-IV	19.03
1422597_at	Mmp15	matrix metallopeptidase 15	10.55
1434734_at	E130016E03Rik	RIKEN cDNA E130016E03 gene	9.11
1441594_at	---	---	5.86
1441624_at	Sorbs2	sorbin and SH3 domain containing 2	3.78
1434028_at	Arnt2	aryl hydrocarbon receptor nuclear translocator 2	11.43
1427967_at	Srgap2	SLIT-ROBO Rho GTPase activating protein 2	9.36
1434497_at	4933431E20Rik	RIKEN cDNA 4933431E20 gene	7.73
1444538_at	Snd1	Staphylococcal nuclease and tudor domain containing 1	8.68
1457636_x_at	---	---	5.12
1436845_at	Axin2	axin2	4.58
1457262_at	2610207I05Rik	RIKEN cDNA 2610207I05 gene	5.35
1431031_at	Arid4b	AT rich interactive domain 4B (Rbp1 like)	3.55
1439380_x_at	Gtl2	GTL2, imprinted maternally expressed untranslated mRNA	7.69
1417520_at	Nfe2l3	nuclear factor, erythroid derived 2, like 3	6.40
1446376_at	Rps6kb1	ribosomal protein S6 kinase, polypeptide 1	5.88
1422882_at	Sypl	synaptophysin-like protein	4.91
1444498_at	---	---	7.23
1438258_at	Vldlr	very low density lipoprotein receptor	6.85
1422954_at	Zfp60	zinc finger protein 60	3.25
1425986_a_at	Dcun1d	DCUN1D1 DCN1, defective in cullin neddylation 1, domain containing 1 (S. cerevisiae)	2.49
1430458_at	Supt6h	suppressor of Ty 6 homolog (S. cerevisiae)	3.17
1434734_at	E130016E03Rik	RIKEN cDNA E130016E03 gene	9.11
1435082_at	Syp1	synaptophysin-like protein	5.30
1437065_at	Zbtb20	zinc finger and BTB domain containing 20	12.39
1441487_at	Trim2	tripartite motif protein 2	5.68
1443628_at	---	---	9.06
1447386_at	Usp2	Ubiquitin specific peptidase 8	2.94
1455327_at	Senp2	SUMO/sentrin specific peptidase 2	4.36

The transcripts shown are significantly altered (2% FDR SAM analysis) exclusively in induced *Cre+Apc*^*fl*/*fl *^*p53*^-/- ^samples compared to induced control *Cre+Apc*^+/+ ^*p53*^+/+ ^samples (not observed in either *Cre+Apc*^*fl*/*fl *^*p53*^+/+ ^or *Cre+Apc*^+/+ ^*p53*^-/- ^compared to control using 2%FDR).

We also employed a candidate gene approach, looking at the expression levels of a number of confirmed Wnt target genes to address whether p53 status affects the amplitude of Wnt signalling following Apc loss. This analysis used Q-RTPCR (Figure 3) to show that a subset of Wnt target genes (CyclinD2, Mash2, Lef1, CD44) are also expressed more highly following Wnt signalling activation in the absence of p53. This is again consistent with a loss of a p53-dependent regulatory feedback loop. Notably, from this analysis it is also clear that some but not all aspects of Wnt signalling are affected by p53 status. For example, Tcf4 (data not shown), Frizzled7 and c-Myc were analysed by Q-RTPCR and showed no differences in expression patterns in the additional absence of p53.

We also employed the candidate gene approach to look at the expression of genes proposed to be involved in the p53 dependent regulatory feedback loops using Q-RTPCR i.e. *p19arf, Mdm2, Siah1a*, *Saih1b *and *Tcf4*. The only noteworthy difference detected was reduced levels of *Siah1b *expression in the induced *Cre+Apc*^*fl*/*fl*^*p53*^-/- ^compared to the induced *Cre+Apc*^*fl*/*fl*^*p53*^+/+ ^samples (Figure 3) (p < 0.05 T-test using ΔC_T _values). Siah1 is a known p53 target gene that appears to be significantly up-regulated (~3 fold up-regulated compared to control, p < 0.05 using T-test of ΔC_T _values) in induced *Cre+Apc*^*fl*/*fl*^*p53*^+/+ ^intestinal samples. Siah1 has previously been shown to interact with APC to promote the degradation of β-catenin, independent of GSK3b phosphorylation of β-catenin [6], and its up-regulation following Apc loss potentially represents a regulatory feedback mechanism. The reduced levels of expression of *Siah1b *in *Cre+Apc*^*fl*/*fl*^*p53*^-/-^compared to *Cre+Apc*^*fl*/*fl*^*p53*^+/+ ^samples confirm p53-dependent expression. However, *Siah1b *expression is still up-regulated (approximately 2 fold) in *Cre+Apc*^*fl*/*fl*^*p53*^-/- ^samples compared to control *Cre+Apc*^+/+^*p53*^+/+ ^samples (Figure 3), suggesting that p53 independent mechanisms also operate to regulate *Siah1b *expression levels following the loss of Apc. Furthermore, the proposed mechanism of *Siah1b *regulation requires an interaction with Apc. However, in our experimental system we have also removed Apc, rendering the proposed *Siah1b *feedback mechanism redundant in this instance.

Finally, we also employed Sam analysis to compare the profiles of induced *Cre+Apc*^*fl*/*fl*^*p53*^-/- ^and induced *Cre+Apc*^*fl*/*fl*^*p53*^+/+ ^intestines. This identified a number of potential differences using an FDR of 15%, (no differences could be identified using a lower FDR) and we subsequently attempted to confirm the expression patterns for a number of genes that the array data suggested were *Cre+Apc*^*fl*/*fl*^*p53*^-/- ^specific alterations. Of 8 transcripts analysed, two (*Slc39a14 *and *Hapb2*) proved to be significantly different between induced *Cre+Apc*^*fl*/*fl*^*p53*^-/- ^and induced *Cre+Apc*^*fl*/*fl*^*p53*^+/+ ^intestines (Figure 3). The significance in the alteration of these 2 transcripts is currently unknown.

## Conclusion

The restricted pattern of p53 up-regulation seen following the loss of Apc (Figure 1) was contrary to expectation, given active Wnt signalling is reported to promote p53 transcription [33]. Our data therefore argues that p53 is not a direct Wnt target, at least in the context analysed here. Notably the upregulation of p53 is associated with a zone of cells showing nuclear atypia, however we show that the development of this atypia is independent of p53, and indeed p21CIP1/WAF1 levels remain high within these cells. Furthermore, our data show that all the immediate phenotypes we observe following Apc loss are unchanged by additional p53 deficiency. With respect to apoptosis, p53 status may be rendered redundant through Wnt-mediated induction of WIP1, a protein which has previously been shown to suppress p53-dependent apoptosis in the intestine [34].

Despite the absence of phenotypic change, deficiency of p53 did modulate the transcriptome. Most significantly, we observed deregulation of a number of key Wnt pathway components and targets. These observations are consistent with a feedback role for p53 in the Wnt pathway; however, this activity is insufficient to modulate the immediate phenotype of Apc loss, possibly because the feedback mechanisms become swamped in the absence of Apc. Overall, our data suggests p53 status has little impact upon the initiating stages of intestinal disease, but they do provide support for physiological Wnt pathway regulation, albeit overwhelmed in this setting. The possibility remains that p53 status truly regulates Wnt pathway activity in settings of lower levels of Wnt signalling. The lack of a potent role for p53 immediately following Apc loss also provides a ready explanation for the association of p53 mutation with only the later stages of colorectal disease [1].

## Competing interests

The authors declare that they have no competing interests.

## Authors' contributions

KRR, VSM, VM and OJS participated in the animal studies; ARC, OJS conceived the study, and KRR, VSM and VM participated in its design and coordination; KRR performed data analysis and presentation; AC performed DNA damage analysis; KRR drafted the manuscript and all authors read and approved the final manuscript.

## Pre-publication history

The pre-publication history for this paper can be accessed here:



## Supplementary Material

Additional file 1 Supplementary table of primers used for QRT-PCR. Click here for file

Additional file 2 P53 IHC on induced *Cre+Apc+/+P53+/+LacZ+*. Click here for file

Additional file 3 Altered probe IDs. Click here for file
