# Supplementary material for: A limited role for p53 in modulating the immediate phenotype of Apc loss in the intestine
Source: BMC Cancer. 2008 Jun 5;8:162. doi: 10.1186/1471-2407-8-162 (PMC2443808; doi:10.1186/1471-2407-8-162)
Supplement: Additional file 1 — Supplementary table of primers used for QRT-PCR. [file 1471-2407-8-162-S1.doc]

Supplementary table of primers used for QRT-PCR

| **Target Gene** | **Forward primer** | **Reverse primer** |
| --- | --- | --- |
| Genes presented in manuscript | | |
| P53 | TGTTATGTGCACGTACTCTCCTC | CTCCGTCATGTGCTGTGACT |
| H2AFX | CCAAGTCGCGCTCTTCAC | AGGATCTCGGCAGTGAGGTA |
| Lect2 | CTGCTCAAAGAACCCAAAGG | CAGACAGTCGAATGCCATCA |
| Axin2 | GCAGCTCAGCAAAAAGGGAAAT | TACATGGGGAGCACTGTCTCGT |
| Mash2 | CAGGAGCTGCTTGACTTTTCCA | GGGCTAGAAGCAGGTAGGTCCA |
| Lef1 | ATTGGCTGGCAAGGTCAG | ATGATGGGAAAACCTGGACA |
| Cd44 | GCCTCAACTGTGCACTCAAA | GTGTTTCAGGGGTGGTCATC |
| CyclinD2 | GGAGAAGCTGTCCCTGATCC | TTCCAGTTGCAATCATCGAC |
| cMyc | CTCAGTGGTCTTTCCCTACCCG3 | TGTCCAACTTGGCCCTCTTGGC |
| Fdz7 | CCATCCTCTTCATGGTGCTT | ATGGCCAAAATGGTGATTGT |
| Siah1b | TGGAAACTTGGGAAGACCAG | CTAGTCGCACGGAGACCTATG |
| Slc39a14 | CTCTCCACGTGCTTTAGCTCT | TCTGGTTTTCTGAGGTGCAG |
| Habp2 | TCATTGGGCTCTCACTGATG | GCAGCTGCAACTGAAGGTATC |
| Genes not presented in manuscript | | |
| Siah1a | TCCGCCCACAGAGATGAG | TGGCCACTCTGACACTGAAG |
| Mdm2 | CAAGAGACTCTGGTTAGACCAAAAC | ACACAATGTGCTGCTGCTTC |
| p19arf | CTGGACCAGGTGATGATGA | ACCAGCGTGTCCAGGAAG |
| Tcf4 | CCGTCCAGGAACTATGGAGA | TTCTGGAATTGACAAAAGGTG |
| Ebf1 | AGCAATGGGATACGGACAGA | GCAATACTCGGCACATTTCA |
| 4933437K13Rik | TGGCTTACTGAGACACTGGATG | CATCTGCTGCACCATAGGAG |
| senp8 | TGAGGAATAGCTTCTGGAATTTCT | CAGCTTGGAGGGTCCAATAG |
| ptprt | CGCAGGTGGCTGTTCTTT | ATTGGCTTCTCCCATGTGTT |
| Slc25a27 | TTACAGACACGTAGTGTACTCTGGA | ATCCCTCCAATGACCGATTT |
| B230114P05Rik | CACAGGAGACCAGGAGAACC | AGGTCTGCGTGGATTCTGAC |
| galnt10 | ACAGCGCGTAGGATATGGAG | ATATCGGGGAGAGAGCGATT |
| nucb2 | GAGCCACCAGATACTGGACTTT | TCCCGCTCCTTATCTCCTCT |
