# Supplementary figures and images for: A limited role for p53 in modulating the immediate phenotype of Apc loss in the intestine
Source: BMC Cancer. 2008 Jun 5;8:162. doi: 10.1186/1471-2407-8-162 (PMC2443808; doi:10.1186/1471-2407-8-162)

## Slide 1
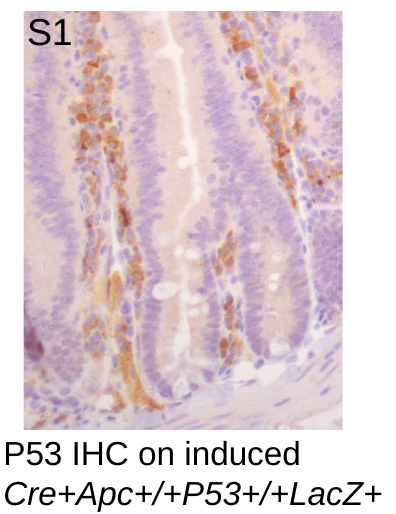

S1
P53 IHC on induced
Cre+Apc+/+P53+/+LacZ+

Supplement: Additional file 2 — P53 IHC on induced Cre+Apc+/+P53+/+LacZ+. [file 1471-2407-8-162-S2.ppt]
