# Supplementary material for: A limited role for p53 in modulating the immediate phenotype of Apc loss in the intestine
Source: BMC Cancer. 2008 Jun 5;8:162. doi: 10.1186/1471-2407-8-162 (PMC2443808; doi:10.1186/1471-2407-8-162)
Supplement: Additional file 3 — Altered probe IDs. [file 1471-2407-8-162-S3.doc]

| Unique to A | Unique to B | Unique to C | Common to A+B | Common to A+C | Common to B+C | Common to A+B+C |
| --- | --- | --- | --- | --- | --- | --- |
| 1426538_a_at  1458366_at  1420816_at  1440207_at  1444350_at  1447960_at  1457619_at  1458225_at  1447665_at  1439580_at  1454873_at  1444280_at  1449832_at  1423008_at  1427536_at  1443960_at  1457790_at  1427514_at  1432353_at  1446876_at  1449602_at  1424190_at  1418173_at  1425552_at  1420007_at  1434634_at  1439654_at  1421453_at  1442401_at  1442433_at  1444665_at  1450014_at  1420718_at  1435420_at  1459132_at  1443621_at  1445245_at  1422618_x_at  1419230_at  1431677_at  1452822_at  1440815_x_at  1435264_at  1460662_at  1419887_at  1436989_s_at  1446987_at  1416286_at  1447523_at  1437572_at  1438012_at  1454579_at  1457379_at  1454587_at  1458031_at  1429317_at  1441638_at  1424808_at  1452300_at  1457110_at  1443554_at  1446740_at  1439972_at  1445008_at  1430609_at  1442885_at  1458419_at  1439290_at  1435924_at  1424601_at  1446015_at  1443087_at | 1445632_at  1428653_x_at  1453789_at  1438757_at  1449391_at  1449913_at  1441997_at  1437737_at  1457351_at  1436648_at  1455400_at  1420856_a_at  1420720_at  1430689_at  1424849_at  1443633_at  1441603_at  1425205_at  1447685_x_at  1442183_at  1442201_at  1442622_at  1443749_x_at  1427658_at  1457971_at  1442721_at  1424566_s_at  1451010_at  1449785_at  1443381_at  1430231_a_at  1458863_at  1431026_at  1451048_at  1443864_at  1443445_at  1445226_at  1416227_at  1441400_at  1429490_at  1418213_at  1459679_s_at  1458318_at  1428069_at  1448482_at  1448780_at  1460021_at  1452665_at  1444602_at  1424784_at  1427376_a_at  1457524_at  1422981_at  1447517_at  1450417_a_at  1454229_a_at  1427776_a_at  1443782_x_at | 1418368_at  1422422_at  1425221_at  1460582_x_at  1421837_at  1422934_x_at  1452183_a_at  1421838_at  1459443_at  1447260_at  1426340_at  1455274_at  1422071_at  1420748_a_at  1423017_a_at  1449492_a_at  1443536_at  1446728_at  1459779_s_at  1421257_at  1446730_at  1459834_x_at  1460604_at  1427580_a_at  1418708_at  1422597_at  1434734_at  1441594_at  1441624_at  1434028_at  1427967_at  1434497_at  1444538_at  1457636_x_at  1436845_at  1457262_at  1431031_at  1439380_x_at  1417520_at  1446376_at  1422882_at  1444498_at  1438258_at | 1423868_at  1424396_a_at  1427499_at  1428371_at  1437141_x_at  1437835_a_at  1440082_at  1440500_at  1446593_at  1450762_s_at | 1422954_at  1425986_a_at  1430458_at  1434734_at  1435082_at  1437065_at  1441487_at  1443628_at  1447386_at  1455327_at | 1417122_at  1417623_at  1418571_at  1418572_x_at  1420978_at  1421237_at  1421682_a_at  1423232_at  1423835_at  1426040_a_at  1429357_at  1435355_at  1438079_at  1438172_x_at  1441178_at  1448147_at  1450128_at  1455521_at  1457689_at  A  72  B  58  C  43  AB  10  ABC  6  AC  10  BC  19 | 1418839_at  1423011_at  1423428_at  1436917_s_at  1449576_at  1450762_s_at |

Data set A are probe ID’s altered in d5 induced *cre^+^Apc^+/+^p53^-/-^* samples compared to d5 induced control *cre^+^Apc^+/+^p53^+/+^* samples using a 10%FDR cut-off by SAM analysis.

Data set B are probe ID’s altered in d5 induced *cre^+^Apc^fl/fl^p53^+/+^* samples compared to d5 induced control *cre^+^apc^+/+^p53^+/+^* samples using a 2%FDR cut-off by SAM analysis.

Data set C are probe ID’s altered in d5 induced *cre^+^Apc^fl/fl^p53^-/-^* samples compared to d5 induced control *cre^+^Apc^+/+^p53^+/+^* samples using a 2%FDR cut-off by SAM analysis.
